# Development of an Epidermal Growth Factor Derivative with EGFR Blocking Activity

**DOI:** 10.1371/journal.pone.0069325

**Published:** 2013-07-30

**Authors:** Clara Panosa, Francesc Tebar, Montserrat Ferrer-Batallé, Humphrey Fonge, Masaharu Seno, Raymond M. Reilly, Anna Massaguer, Rafael De Llorens

**Affiliations:** 1 Biochemistry and Molecular Biology Unit. Department of Biology, University of Girona, Campus Montilivi, Girona, Catalunya, Spain; 2 Department of Cell Biology, Immunology and Neurosciences, University of Barcelona, Barcelona, Catalunya, Spain; 3 Department of Pharmaceutical Sciences, Leslie Dan Faculty of Pharmacy, University of Toronto, Toronto, Ontario, Canada; 4 Department of Biotechnology, Graduate School of Natural Science and Technology, Okayama University, Okayama, Japan; 5 Toronto General Research Institute, University Health Network, Toronto, Ontario, Canada; University of Central Florida, United States of America

## Abstract

The members of the epidermal growth factor (EGF)/ErbB family are prime targets for cancer therapy. However, the therapeutic efficiency of the existing anti-ErbB agents is limited. Thus, identifying new molecules that inactivate the ErbB receptors through novel strategies is an important goal on cancer research. In this study we have developed a shorter form of human EGF (EGFt) with a truncated C-terminal as a novel EGFR inhibitor. EGFt was designed based on the superimposition of the three-dimensional structures of EGF and the Potato Carboxypeptidase Inhibitor (PCI), an EGFR blocker previously described by our group. The peptide was produced in E. coli with a high yield of the correctly folded peptide. EGFt showed specificity and high affinity for EGFR but induced poor EGFR homodimerization and phosphorylation. Interestingly, EGFt promoted EGFR internalization and translocation to the cell nucleus although it did not stimulate the cell growth. In addition, EGFt competed with EGFR native ligands, inhibiting the proliferation of cancer cells. These data indicate that EGFt may be a potential EGFR blocker for cancer therapy. In addition, the lack of EGFR-mediated growth-stimulatory activity makes EGFt an excellent delivery agent to target toxins to tumours over-expressing EGFR.

## Introduction

The cells of multi-cellular organisms need a constant communication with the environment to maintain their homeostasis, to survive, to differentiate, and to proliferate. One of the most important communication systems is represented by the Polypeptide Growth Factors that include eight families. The Epidermal Polypeptide Growth Factor (EGF)/ErbB family represents a very important – in fact essential – family of growth factors governing a multitude of cellular events. Its importance is also reflected by its crucial role in many pathologies, most notably in cancer where they are involved in the sustained chronic proliferation of cancer cells [1]. The prominent role of the ErbB family in the development and surviving of cancer cells was described in the 1980's, when Sporn and Todaro established the theory of the “*autocrine secretion*” of growth factors by cancer cells to maintain a high rate of proliferation [2]. In many types of cancer the over-expression of ErbB receptors and enhanced ligand production allow an autocrine stimulation of the cell proliferation. Since then an enormous amount of literature has been devoted to the role of the receptors, but to a lesser extent to the ErbB ligands. After decades of high interest in the ErbB receptors as therapeutic targets, and low attention focused on the ligands, new insights have renewed the importance of the ErbB ligands, especially the EGFR ligands, as responsible for autocrine/paracrine loops and resistance to old and new therapeutic agents [3]–[6]. We would like to suggest that “it is time to turn again our attention to the role played by the ligands”, not only the receptors, to design new therapeutic strategies against cancer.

**Figure 1:**
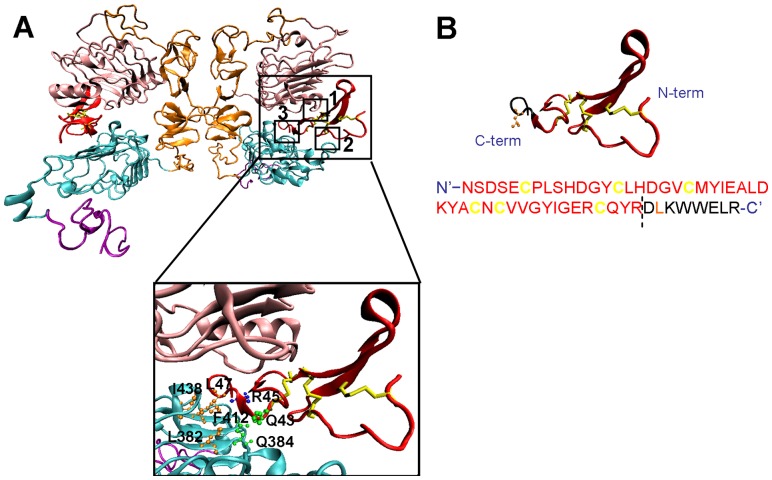
Ribbon diagram of the crystal structure of an EGFR homodimer in complex with two EGF ligands (from pdb code 1IVO). A The subdomains I, II, III and IV of both receptors are colored pink, orange, cyan and purple, respectively. Held between domains I and III of each receptor are the EGF ligands colored red and their disulfide bridges yellow. The three main interactions sites between EGF and EGFR are outlined (1, 2 and 3). The interaction site 3 between C-terminal part of EGF and domain III of EGFR is magnified. This binding site is composed by two different kinds of interactions: hydrophobic interactions between Leu47 (EGF) and Leu382, Phe412, and Ile438 (EGFR), and the formation of hydrogen bonds with Gln384 side chain of EGFR and carbonyl and amide groups of Gln43 and Arg45, respectively, of EGF. All side chains of residues responsible for these interactions are shown in different colors depending on the type of interaction. B Ribbon diagram and amino acid sequence of EGF. The C-terminal part of the molecule is highlighted in black and the side chain of Leu47 is shown in orange in the ribbon diagram. The nucleotides that encode these 8 amino acids were removed from the encoding sequence to construct the EGF truncated form (EGFt).

Human EGF (hEGF) is a small single chain polypeptide composed of 53 amino acids that adopts a well-defined three dimensional structure containing three disulfide bonds defining three loops (namely A, B and C). This scaffold is known as a T-Knot and is found in a large number of functional unrelated proteins [7]. EGF structure is tightly related to its function. There are three contact sites described between the ligand and the EGFR which in turn is divided by four domains (I, II, III and IV) [8] (Figure 1): the B loop of EGF interacts with site 1 in domain I, the A loop of EGF interacts with site 2 in domain III, and the C-terminal region of EGF interacts with site 3 in domain III. This structure–function relationship in EGF has been accurately examined in order to generate antagonistic analogues of EGF, or EGFR blockers. Some authors have developed shorter synthetic derivatives of EGF and TGF-α as antagonists of these growth factors. However, these strategies have not been effective because the EGF and TGF-α fragments were too short to form the tertiary structure required for binding to the EGFR [9]. In other studies the EGF derivative required very high concentrations to exhibit the desired responses, probably because the short peptide interacted only with one of the three interfaces of interaction, namely the site 1 of EGFR [10].

Our group has previously described that the Potato Carboxypeptidase Inhibitor (PCI) is an antagonist of human EGF. PCI is a 39-residue protein folded into a 27-residue globular core stabilized by three disulfide bonds that adopts the same conformation as EGF, the T-Knot scaffold. This similar structure probably accounts for the antagonistic activity. PCI competed with EGF for binding to EGFR, inhibited EGFR activation and cell proliferation and induced the down regulation of the receptor. In addition, PCI showed anti-proliferative activity *in vitro* in several human cancer cell lines and *in vivo* in nude mice implanted with a xenograft of pancreatic cancer cell lines [11], [12]. Unfortunately, PCI's affinity for EGFR is very low, and high concentrations were required to achieve the desired inhibitory activity. The structural and clinical interest of PCI opened the possibility of engineering new PCI-like EGF antagonists with improved ErbB affinity. The superimposition of the three-dimensional structures of EGF and PCI based on disulfide bridge topology [7], [11], [12], revealed that PCI lacks the C-terminal part of EGF, one of the important sites described for interaction with domain III of the receptor. Our hypothesis was that the lack of this interaction could be responsible for the described anti-proliferative properties of PCI since EGFR needs a ligand that binds with high affinity to both domains I and III to activate the intracellular signalling pathway that promotes cell division [13], [14].

In the present study we describe the production of the human EGF derivative, EGFt, which lacks the eight final C-terminal amino acids of EGF in order to preserve the EGF high affinity for the receptor and the anti-tumour properties of PCI. We describe the differences between the activation of EGFR by wild type hEGF or by EGFt, focusing on the key steps of activation pathway: ligand binding, dimerization, trans-phosphorylation and internalization. We also compare the cell growth promoting effect of both peptides in order to evaluate the potential application of EGFt as EGFR blocker for cancer therapy as well as a potential delivery agent of radio-nuclei conjugates or toxins to cancer cells overexpressing EGFR.

## Materials and Methods

### Materials

Restriction enzymes and T4 DNA ligase were all purchased from New England Biolabs (Ipswich, MA, USA). High-Fidelity DNA polymerase was from Finnzymes (Espoo, Findland). Commercial recombinant human Epidermal Growth Factor and tangential flow filter were purchased from Millipore (Massachusetts, USA). The anionic exchange column for HPLC was a HiPrep DEAE FF 16/10 and the gel filtration HPLC column was a Superdex 75 10/300 GL, both from GE Healthcare Bio-Sciences AB (Uppsala, Sweden). Reverse-phase HPLC column was a Vydac 218TP C18 from Grace (Illinois, USA).

### Molecular cloning and expression of hEGF and EGFt on small batch culture conditions

hEGF and EGFt encoding sequences were synthesised by GenScript (Piscataway, NJ, USA). The sequences included the *ompA* leader sequence and the enzyme restriction site *XbaI* on the 5′ end of the encoding sequences. Next, the sequences were digested with the *XbaI* and *BamhI* restriction enzymes and ligated to the expression vector pIN-III-ompA-2 [15] using T4 DNA ligase. The constructed plasmids, ompA_hEGF and ompA_EGFt, were transformed into *E. coli* MC1061 (kindly donated by Dr. Querol, Autonomous University of Barcelona, Spain). The positive clones were identified by diagnostic restriction enzyme digestion and then sequenced to confirm the correct hEGF and EGFt cloning (ABI PRISM 310 Genetic Analyzer, Applied Biosystems (Foster City, CA, USA)). *E.coli* MC1061 cells containing ompA_hEGF or ompA_EGFt plasmids were grown at 37°C in 400 ml culture of M9 minimal medium supplemented with casamino acids (M9 CAS) and with 50 µg/ml ampicillin (Sigma-Aldrich, St. Louis, MO, USA). After induction with 0.2 mM isopropyl β-D-thiogalactopyranoside (IPTG) (Sigma-Aldrich) cells were incubated for 24 hours. Next, to determine the presence of the peptides in the extracellular fraction, the culture were harvested by centrifugation at 15,000×g for 15 min and immediately subjected to cellular fractionation to obtain the different fractions of the culture [16].

### Production of hEGF and EGFt in a 10 liter fermentor

OmpA_hEGF or ompA_EGFt MC1061 *E.coli* positive colonies were added into a 100 ml Luria-Bertani medium (LB) supplemented with 50 µg/ml ampicillin. The culture was grown at 37°C in a rotary shaker (250 rpm) for a minimum of 5 hours. Then, 5 mL of the culture were inoculated into a flask with 300 mL M9 CAS medium supplemented with 50 µg/ mL ampicillin and grown at 37°C, over night, in a rotary shaker (250 rpm). The entire 300 ml grown culture was inoculated in 5000 ml of the same medium in a 10-L fermentor. The temperature was maintained at 37°C, the partial pressure of O_2_ at 65% and the pH at 7. The culture was continuously fed from the 2 early hours of the fermentation to the 6 h hours with 50% of casamino acids and glycerol. 1–2 hours later M9 salts (33% of the total volume) and 0.4 mM of IPTG were added into the cell culture. After that, the culture was continuously fed with M9 salts, oligoelements, and the other 50% of casaminoacids and glycerol, overnight. 24 hours later the culture medium was collected. The fermentation was carried out by the Fermentation Service of the University of Barcelona using a Biostat B 10L laboratory fermenter (Sartorius BBI Systems, Postfach, Melsungen, Germany).

### Protein purification

The culture medium obtained either by flask or by fermentation was centrifuged at 10000×*g* and 4°C for 30 min, twice. The supernatant corresponding to the extracellular medium was then filtered through 0.4 and 0.2 µm membranes, successively, and concentrated by tangential flow filtration using a 3000-Da membrane. The concentrated sample was then dialysed against 50 mM Tris-HCl pH 7.5 (solvent A) to subsequently perform HPLC (GE Healthcare Bio-Sciences AB: ÄKTAbasic) using an anionic-exchange column (HiPrep DEAE FF 16/10)). The flow rate was 1 ml/min and the elution buffer (solvent B) was: 50 mM Tris-HCl, 1M NaCl at pH 7.5. The samples were eluted with a linear gradient from 0–10% solvent B for 40 min, 10–30% for 80 min and 30–50% for 40 min. Samples containing the recombinant protein were eluted and collected after 60 min of elution and lyophilised. In the last purification step, the sample was dissolved with elution buffer (50 mM Tris-HCl, 200 mM NaCl at pH 8) and then separated by a gel-filtration HPLC chromatography column at a flow rate of 1.2 ml/min. The recombinant proteins were eluted after about 13 min of elution time.

### Protein analysis

The presence of hEGF or EGFt in each production step was determined on a 16% sodium dodecylsulfate–polyacrylamide gel electrophoresis (SDS-PAGE). The gel was stained with Coomassie brilliant blue or analyzed by Western blotting using a primary rabbit polyclonal antibody against human EGF (Z-12) (sc-275) (Santa Cruz Biotechnology, Santa Cruz, CA, USA) and a secondary antibody coupled to horseradish peroxidase (Dako, Glostrup, Denmark). The total protein quantity present in the samples was determined by Lowry-based Bio-Rad assay (Bio-Rad Laboratories, Hercules, CA, USA). The identity and purity grade of the proteins were assessed by MALDI-TOF MS and N-terminal amino acid sequencing (Proteomics and Bioinformatics facility from Autonomous University of Barcelona, ProteoRed network). The state of folding of the EGF peptides was analyzed by reverse phased high performance liquid chromatography (RP-HPLC) using the methodology described by Chang et al. [17], [18] that allows the identification of minute concentrations of non-native disulfide scrambling isomers.

### Cells and cell culture

The human colorectal adenocarcinoma cell line Caco-2 and the breast adenocarcinoma cell lines MDA-MB-468 and MCF-7 were obtained from the American Type Culture Collection (Manassas, VA, USA). The cells were maintained in Dulbecco's modified Eagle's medium supplemented with 10% fetal bovine serum (FBS) and 1% penicillin-streptomycin (Gibco-BRL, Grand Island, NY, USA) at 37°C in a humidified atmosphere containing 5% CO_2_. Cell growth and morphology were assessed daily using an inverted microscope. The cells were routinely maintained for up to 8 passages by successive trypsinization and seeding and the cell viability was assessed by trypan blue staining (only cultures displaying >90% viability were used for further work). Possible contamination with Mycoplasma was routinely checked using the VenorH GeM Mycoplasma Dection Kit (Minerva Biolabs GmnH, Berlin, Germany).

### Immunofluorescence analysis

The quantification of EGFR expression was performed by flow cytometry. The cells were analyzed by double immuno-fluorescence using a mouse monoclonal antibody against human EGFR (GR13 Anti-EGFR (Ab-3)), (Calbiochem, San Diego, CA, USA). The cells were incubated for 30 min at 4°C with the primary antibody or an irrelevant antibody as negative control. After washing with phosphate-buffered saline (PBS) (Gibco-BRL), the cells were incubated for an additional 30 min at 4°C in the presence of the Alexa-Fluor 488-conjugated goat anti-mouse IgG antibody (Invitrogen, Carlsbad, CA, USA). Next, the fluorescence was analyzed using a FACSCalibur flow cytometer (Becton Dickinson Immunocytometry Systems, San Jose, CA, USA) equipped with CellQuest™ software (Becton Dickinson). Fluorescence intensity was represented on a four orders of magnitude log scale (1–10,000). In each experi­ment 10,000 cells were analyzed.

### Receptor binding assay

EGFt was derivatized with diethylenetriamine pentaacetic acid (DTPA) (Sigma-Aldrich) and radiolabeled with ^111^Indium-acetate (Nordion, Kanata, ON, Canada) to a specific activity of 3.7–18.5 MBq/μg as described [19]. The radiochemical purity of ^111^In-labeled EGFt was 95%–98% as assessed by silica gel instant thin layer chromatography in 100 mmol/L sodium citrate (pH 5). The receptor-binding properties of ^111^In-labeled EGFt were evaluated in a direct radioligand binding assay using MDA-MB-468 human breast cancer cells (1x10^6^ EGFR/cell [19]). Briefly, various concentrations of ^111^In-labeled EGFt (0.6 nM-300 nM) in 120 μL of 150 mM NaCl containing 0.2% bovine serum albumin (BSA) (Roche, Penzberg, Germany) were incubated with 1x10^6^ cells in 1.5 mL microtubes for 3 h at 4°C. Cell bound radioactivity was separated from free radioactivity by centrifugation at 2,700×g for 5 min, and then counted in a γ-scintillation counter (Perkin Elmer, Shelton, CT, USA). Non-specific binding was determined by conducting the assay in the presence of an excess (30 μM) of unlabeled EGFt. Specific binding was obtained by subtraction of non-specific binding from total binding. The equilibrium dissociation constant value (K_d_) was estimated by nonlinear regression of a plot of the specific binding versus the concentration of ^111^In-DTPA-EGFt incubated with the cells using GraphPad Prism software [20]. The K_d_ value of ^111^In-DTPA-hEGF was obtained from previous work [21].

### Western blot analysis

Cells were collected and lysed with ice-cold lysis buffer containing 20 mM sodium phosphate pH 7.4; 150 mM NaCl; 1% Triton X-100; 5 mM EDTA; 5 mM PMSF; 10 μg/ml aprotinin and leupeptin; 250 μg/ml sodium vanadate (Sigma-Aldrich). Protein concentrations were determined by Lowry-based Bio-Rad assay (Bio-Rad Laboratories, Hercules, CA, USA). Equal amounts of protein extracts were electrophoresed on an 8% SDS-PAGE gel, transferred to a polyvinylidene difluoride membrane (Millipore, Bedford, MA, USA) for 3 hours at 100V and then blocked for 1 h h at room temperature in a blocking buffer containing 2.5% powdered skim milk in PBS-T (10 mM Tris-HCl, pH 8.0, 150 mM NaCl and 0.05% Tween-20) to prevent non-specific antibody binding. Blots were incubated overnight at 4°C with the corresponding primary antibody diluted in blocking buffer. After washes with PBS-T, blots were incubated for 1 h with the corresponding secondary antibody coupled to horseradish peroxidase (Dako), and revealed with a commercial kit (West Pico Chemiluminescent Substrate, Pierce Biotechnology, Rockford, IL, USA). Blots were reprobed with an antibody for β-actin (Santa Cruz Biotechnology, CA, USA) to control the protein loading and transfer.

### Dimerization analysis

Chemical cross-linking experiments were designed to examine the ability of hEGF and EGFt proteins to induce receptor dimerization. The protocol for cross-linking was previously described by Canals F et al. [22]. MDA-MB-468 cells were seeded in 100-mm dishes and allowed to grow to 50% confluence. Cells were harvested in 0.4 ml ice-cold lysis buffer (20 mM sodium phosphate pH 7.4; 150 mM NaCl; 1% Triton X-100; 5 mM EDTA and protease and phosphatase inhibitor cocktails (Sigma-Aldrich)). The protein concentration in cell lysates was determined by a Lowry-based Bio-Rad assay (Bio-Rad Laboratories, Hercules, CA, USA). Aliquots of 10 μg of protein were stimulated with 150 nM hEGF or 150 nM and 15 μM EGFt, and additionally 150 nM hEGF together with 150 nM EGFt, at room temperature. After 30 min incubation, EGFR cross-linking was induced by addition of 40 mM glutaraldehyde. 1 min later the reaction was stopped with 0.2 M glycine (pH 9). Next, loading buffer with 5% β-mercaptoethanol was added to the samples and heated for 5 min at 100°C. The samples were electrophoresed on 5% polyacrylamide gels (SDS-PAGE), transferred onto PVDF membranes at 30V overnight at 4°C and analysed by Western blotting as described. Rabbit polyclonal antibodies against human EGFR (EGFR 1005 (sc-03)) and against HER2 (Neu (C-18)), (both from Santa Cruz Biotechnology) were used as primary antibodies.

### Analysis of EGFR activation and degradation

The ability of EGFt to activate the total phosphotyrosines of the receptor was determined in MDA-MB-468 cells after treating the cells with 3 nM, 150 nM hEGF or 150 nM EGFt for 15 minutes at 37°C. Next, the same amount of cell lysates were analyzed in parallel by Western blotting with a mouse monoclonal antibody against total phosphotyrosines conjugated to horseradish peroxidase (PY20) (sc-508 HRP, Santa Cruz Biotechnology) and a primary antibody against EGFR (EGFR 1005). EGFR specific phosphoresidues were examined in MDA-MB-468, MCF-7 and Caco-2 cells after treating the cells with 150 nM hEGFR or EGFt at 37°C for different incubation times. Samples were analysed by Western blotting using primary monoclonal antibodies against phospho-EGFR Tyr 1068 (#2236), Tyr 1173 (#4407), Tyr 1045 (#2237) and Ser 1046/47 (#2238) (Cell Signaling Technology, Danvers, MA, USA). MAPK and AKT activation was analyzed with polyclonal antibodies against phosphorylated MAPK (ERK1/2) and phospho-Akt (Thr308), both from Cell Signaling, New England Biolabs (Beverly, MA).

For the analysis of EGFR degradation Caco-2 and MCF-7 cells were incubated at 37°C with starvation medium containing 10 μg/mL of cycloheximide (Sigma Aldrich) and 3 nM, 150 nM hEGF or 150 nM EGFt for different times. After treatments, cells were collected and lysed and the different samples were analysed by Western blotting using the anti-EGFR antibody (EGFR 1005).

### EGFR internalization analysis

The capacity of hEGF and EGFt to induce the internalization of EGFR was determined by a cell-ELISA assay. MCF-7 and Caco-2 cells were seeded in 96-well culture plates (Costar 3596; Corning Inc., Corning, NY, USA) at a density of 4000 and 5000 cells/well, respectively. The cells were allowed to attach and grow until they reached 70% of confluence and then 3 nM hEGF, 150 nM hEGF, 150 nM EGFt, or PBS (control cells) were added. Next, the cells were incubated for 30 min at 4°C to allow the binding of EGF to its receptor and then 15 min at 37°C to allow internalization. The cells were subsequently washed with PBS, fixed with 2% formaldehyde for 20 min at room temperature and blocked with 1% BSA in PBS for 1 h at room temperature. After removal of the blocking solution, the plates were incubated for 1 h at room temperature with the anti-hEGFR mouse monoclonal antibody (GR13 (Ab-3)) (Calbiochem). The samples were subsequently washed and incubated for 1 h h with a peroxidase-conjugated secondary antibody (Polyclonal Goat Anti-Mouse Immunogulobulins/HRP; Dako, Glostrup, Denmark). Briefly, the plates were washed with PBS, and 100 μl of 3,3′-5,5′-tetramethyl benzidine (TMB) (Roche, Penzberg, Germany) solution was added to each well according to the manufacturer's directions. The plate was incubated for 5 min at room temperature and then the enzymatic reaction was stopped by the addition of 100 μl of 1 M H_2_SO_4_. The absorbance at 450 nm was measured with a microplate reader (ELx800; BioTek, Winooski, VT, USA). Each experiment, including untreated control cells, was carried out in triplicate and the mean absorbance for each treatment was calculated. The EGFR present on the cell surface was determined as a percentage of untreated control cells by dividing the mean absorbance of the three replicates by the mean absorbance of the untreated control cells.

### Immunofluorescence staining and confocal microscopy

MDA-MB-468 cells grown on coverslips were treated with 150 nM of hEGF or EGFt for different time periods and then fixed with freshly prepared 4% para-formaldehyde (Electron Microscopy Sciences, Hatfield, PA, USA) for 12 min at room temperature and mildly permeabilized with PBS containing 0.1% Triton X-100 and 0.1% BSA (Roche, Penzberg, Germany) for 3 min at room temperature. Coverslips were then incubated in the same buffer, in which Triton X-100 was omitted, at room temperature for 1 h with the anti-hEGFR monoclonal antibody (GR13 (Ab-3)) (Calbiochem) and a monoclonal antibody against the nuclear membrane marker Lamin-B1 (A-11) (sc-377000) (Santa Cruz Biotechnology). Coverslips were washed intensively, and then incubated with adequate secondary antibodies labelled with Alexa Fluor 488 and Alexa Fluor 555 (Invitrogen, Carlsbad, CA, USA), respectively. Both primary and secondary antibody solutions were precleared by centrifugation at 14,000×*g* for 10 min. Hoechst (Invitrogen, Carlsbad, CA, USA), was incubated together with the secondary antibodies to stain the cell nucleus. Next, the coverslips were mounted in Mowiol mounting medium (Calbiochem, San Diego, CA, USA). The images were obtained using a Leica TCS SPS laser-scanning confocal spectral microscope (Leica Microsystems, Wetzlar, Germany). Final analysis of deconvolved images was performed using ImageJ software (http://rsb.info.nih.gov/ij/).

### Cell proliferation assay

To assess the ability of hEGF and EGFt to promote cell-growth, Caco-2 and MCF-7 cells were seeded in 96-well plates (5000 and 4000 cells/well, respectively). The optimal cell seeding concentration was previously established for both cell types, as described in the supporting information (Fig S1), in order to maintain the cells in an exponential growth phase along the experiment. The cells were allowed to attach and grow for 72 h in culture medium, then the cells were washed and medium without FBS was added to the cells for 24 h. Next, the cells were treated with concentrations ranging from 0 to 150 nM of hEGF or EGFt for 96 h for Caco-2 cells and 72 h for MCF-7 cells. The treatments were removed, and the cells were washed with PBS and incubated for 3 h with 100 μl of fresh culture medium together with 10 μl of MTT (3-(4,5-dimethylthiazol-2-yl)-2,5-diphenyltetrazolium bromide) (Sigma-Aldrich, St. Louis, MO, USA). Medium was discarded and dimethyl sulfoxide (DMSO) (Sigma-Aldrich, St. Louis, MO, USA) was added to each well to dissolve the purple formazan crystals. Plates were agitated at room temperature for 10 min and the absorbance of each well was determined with an absorbance microplate reader (ELx800, BioTek, Winooski, VT, USA) at a wavelength of 570 nm. Three replicates were used for each treatment. The cell viability was determined as a percentage of the untreated control cells, by dividing the mean absorbance of each treatment by the mean absorbance of the untreated cells.

### Statistical analysis

Statistical analysis was performed with the SPSS statistical software for Windows (version 15.0; SPSS Inc., Chicago, IL, USA). Quantitative variables were expressed as mean and standard error (SE). The normality of the data was tested using the Kolmogorov-Smirnov test. The differences between data with normal distribution and homogeneous variances were analyzed using the parametric Student's t-test, otherwise the non-parametric Mann-Whitney U test was applied. A value of P<0.05 was considered significant.

## Results

### Design of an EGF analogue

Figure 1A shows the ribbon diagram of the crystal structure of an EGFR homodimer in complex with two EGF ligands. The three main interaction sites between EGF and EGFR as well as the specific residues in site 3 involved in the interaction between the receptor and EGF are indicated. Based on this structure we designed a truncated EGF analogue, EGFt, lacking the 8 C-terminal amino acids, including the Leu47 residue important for the hydrophobic interactions in site 3, in order to generate a peptide with blocking activity and higher affinity than PCI for the receptor. In figure 1B the ribbon diagram and the amino acid sequence of EGF are represented, with the removed C-terminal aminoacids depicted in black and the Leu47 residue depicted in orange.

### Production, purification and analysis of recombinant hEGF and EGFt

Wild type hEGF was produced in parallel to EGFt in order to obtain a positive control for the experiments and for future applications. The encoding sequences of hEGF and EGFt were cloned into a periplasmic secretion system that contains the OmpA (Outer membrane protein A) signal sequence [23] in order to obtain the peptides in the supernatant of the cell culture. After 24 h of culture we confirmed by cell fractionation that hEGF and EGFt were mainly in the extracellular fraction of the cell culture (data not shown). The yield of production of these proteins on a shake flask level was about 1 mg/L of culture. To improve this yield, the production of the recombinant proteins was next conducted in a Biostat B 10 L laboratory fermenter under fed-batch fermentation conditions and the production increased by 25-fold.

**Figure 2:**
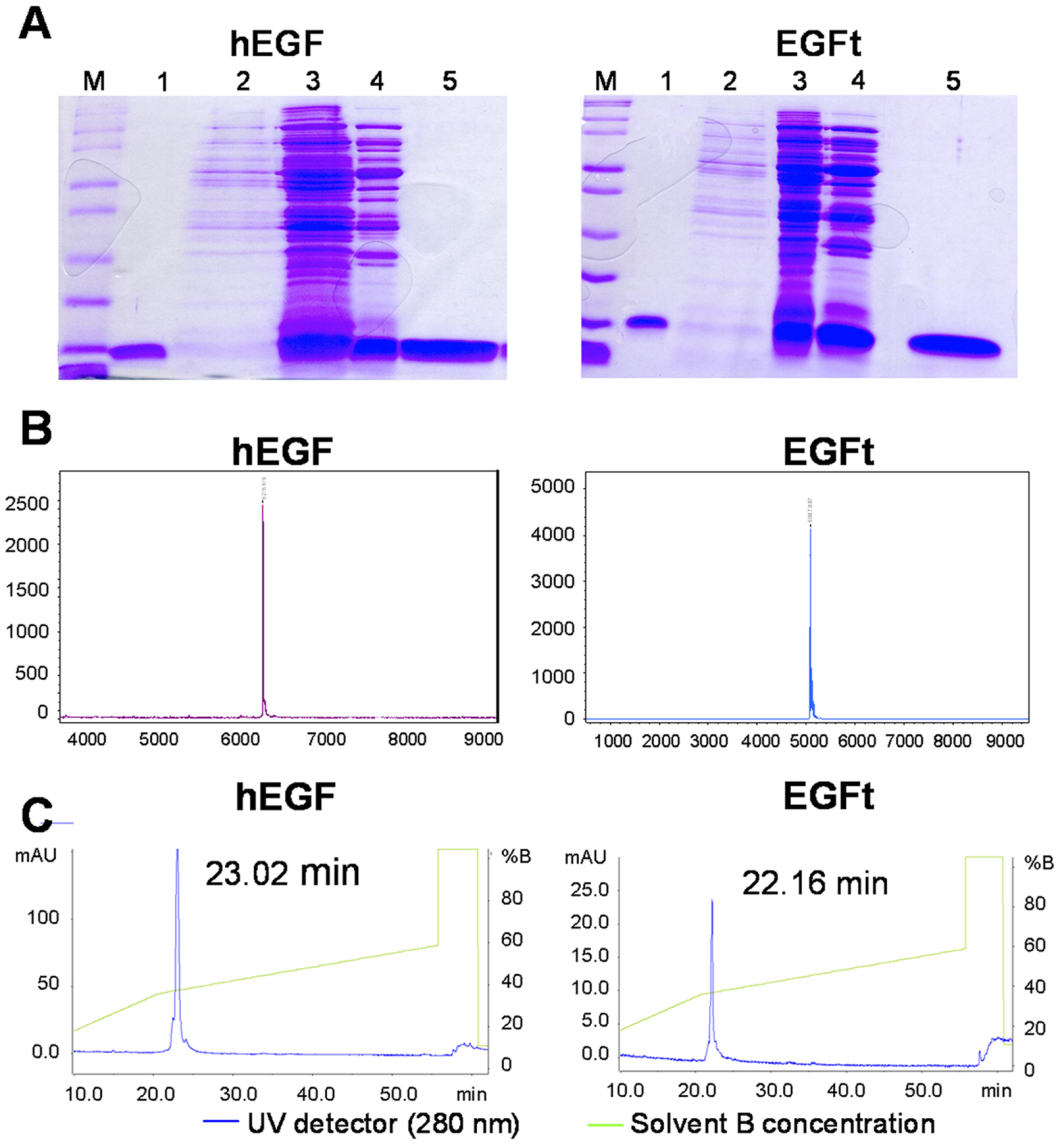
Purification and characterization of hEGF and EGFt. A Protein analysis of the different purification steps by Coomassie blue-stained SDS-PAGE. Molecular weights (M); Lane 1: commercial recombinant hEGF. Lane 2: supernatant of the *E.coli* fermentor culture before concentration. Lane 3: 30x concentrated supernatant by tangential flow filtration. Lane 4: product of the first purification step (anionic exchange chromatography). Lane 5: purified product after the final purification step (gel filtration chromatography). B Determination of the molecular weight of hEGF and EGFt by mass spectrometry (MALDI-TOF). The analysis confirmed the corresponding molecular weight of the hEGF (6216.6 Da) and EGFt (5087.9 Da). C Analysis of the state of folding of purified hEGF and EGFt by RP-HPLC. The peaks on the chromatogram correspond to the elution time of well-folded hEGF.

Next, the proteins were concentrated from the supernatant and purified by two chromatographic steps by HPLC: anion exchange and size exclusion chromatography. Fig. 2A shows the SDS-PAGE analysis of the samples obtained in the different purification steps confirming the protein purity in the last step for both peptides. The identities of both proteins were assessed by MALDI-TOF analysis confirming their high purity and expected molecular mass: 6222 Da for hEGF and 5094.7 Da for EGFt (Fig. 2B). Additional, N-terminal amino acid sequence analysis confirmed the correct amino acid sequence and the proper cleavage of the ompA signal peptide in the periplasm (data not shown). Fig. 2C shows the protein folding analysis by reverse-phase HPLC. Both chromatograms contain a single peak at the retention time described for hEGF indicating its correct folding and the absence of folding scrambles [18].

### EGFR expression profile in three EGFR-positive human tumor cell lines

**Figure 3:**
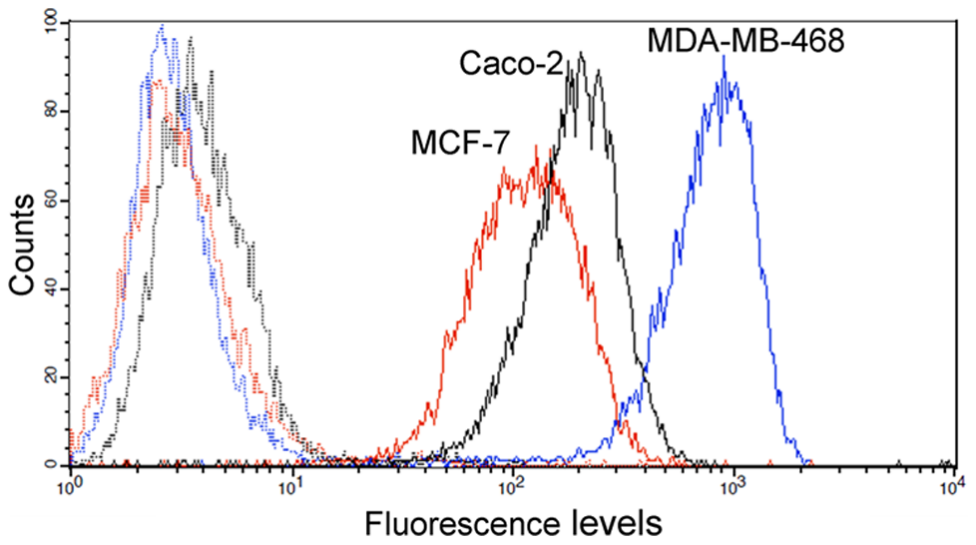
EGFR expression profile in MDA-MB-468, MCF-7 and Caco-2 cells. The expression of EGFR on the cell surface was determined by flow cytometry after indirect immunofluorescence staining with a monoclonal antibody against human EGFR (solid lines) followed by incubation with fluorescent-labelled secondary antibody. An irrelevant primary antibody was used as a negative control (dotted lines). Histograms were obtained after analyzing 10,000 MCF-7 (red), Caco-2 (black) and MDA-MB-468 (blue) cells. The fluorescence intensity is shown on a four-decade log scale.

Before conducting the biological assays, we examined the EGFR expression levels in MDA-MB468, MCF-7 and Caco-2 cells, with a well-described autocrine loop of TGF-α, by flow cytometry. As represented in Fig. 3, MDA-MB-468 cells expressed the highest level of EGFR, while MCF-7 and Caco-2 cells showed similar moderate expression of EGFR.

### EGFR binding affinity of EGFt

**Figure 4:**
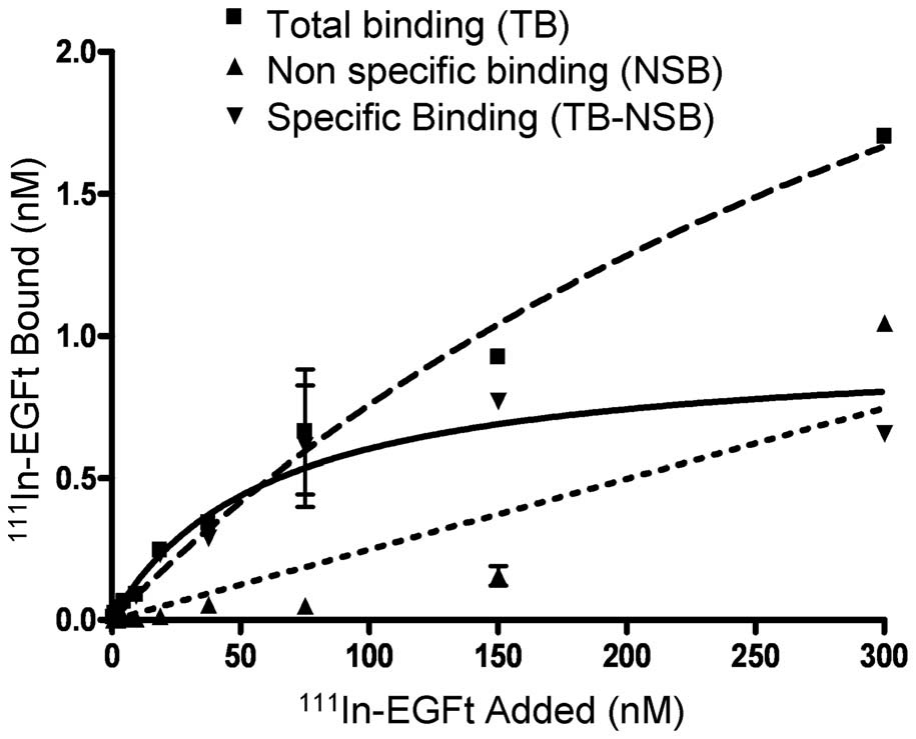
Representative binding curve for ^111^In-DTPA-EGFt to MDA-MB-468 cells. The total binding curve was obtained by incubating cells with increasing concentration of ^111^In-labeled EGFt (0–300 nM). The non-specific binding curve was obtained by adding increasing concentrations of the ^111^In-DTPA-EGFt in the presence of 100-fold excess of unlabeled EGFt (30 μM). The specific binding curve was obtained by subtracting non-specific binding from total binding. The dissociation constant (K_d_) for ^111^In-labeled EGFt measured in this assay was 60.05±2.03 nM. Each point represents the mean ± SEM of 3 assays performed in triplicate.

MDA-MB-468 cells were used to analyze the binding affinity of the truncated EGF analogue for EGFR. In Fig. 4, the total, specific and non-specific binding of ^111^In-DTPA-EGFt has been plotted against increasing concentrations of radiolabelled ligand. The K_d_ for specific binding of ^111^In-DTPA-EGFt was calculated using a one-site binding hyperbola nonlinear regression analysis by GraphPad Prism software and indicated that the receptor-binding affinity of ^111^In-DTPA-EGFt for EGFR (K_d_  =  6±2.03 M×10^−8^) was approximately 46-fold lower than that of ^111^In-DTPA-hEGF (K_d_  = 1.3±0.26 M×10^−9^). For the following experiments, we decided to use 150 nM nM of hEGF and EGFt, as a saturating concentration. In some experiments 3 nM of hEGF was also included since it is the equivalent concentration of the 150 nM EGFt, based on its binding affinity to the receptor.

### EGFR dimerization

**Figure 5:**
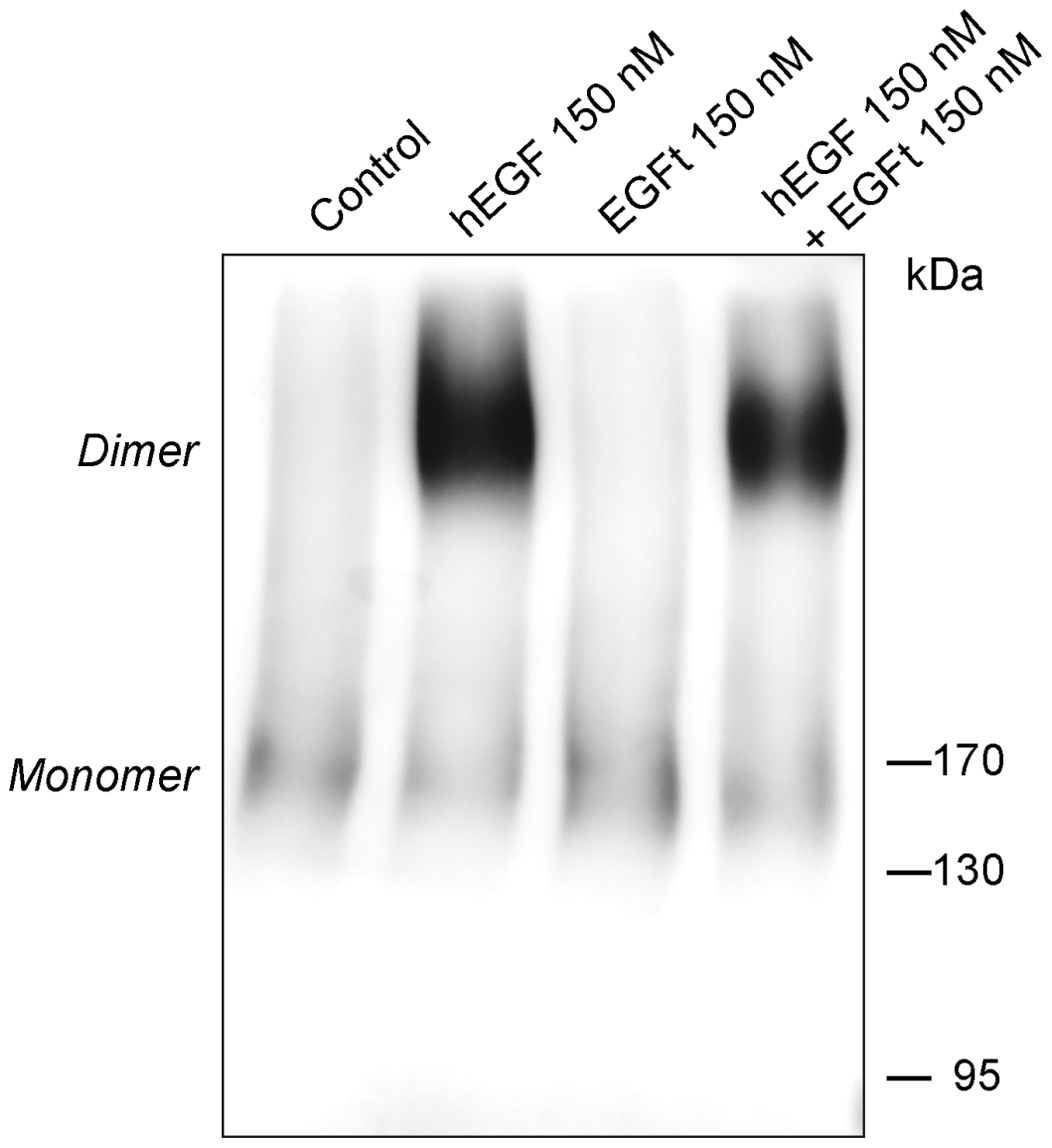
Effect of EGFt on the dimerization of EGFR. Cell lysate from MDA-MB-468 cells were treated with the indicated concentrations of hEGF, EGFt or a mixture of both for 30 min. Then the samples were cross-linked by addition of 40 mM of glutaraldehyde and analyzed by Western blotting using an anti-EGFR antibody. The position of the EGFR monomers and dimmers is indicated.

MDA-MB-468 cell lysates were treated with either hEGF or EGFt and in combination for 30 min and then the proteins were cross-linked by adding 40 mM of glutaraldehyde. The dimer formation was analyzed by Western blotting. As expected, hEGF induced EGFR dimers, while no dimerization of EGFR could be detected after the EGFt treatment (Fig. 5), even when the concentration was increased to 15 μM (Fig. S2A). These results suggest that the homodimers were not effectively induced or stabilized by EGFt stimulation. Moreover, when hEGF and EGFt were mixed at the same concentration, the EGFR dimer formation decreased compared to hEGF treatment (Fig. 5), indicating that EGFt was competing with hEGF for EGFR binding and competitively decreasing dimer formation. EGFt could neither induce EGFR-HER2 heterodimerization (Fig. S2B).

### EGFR activation

**Figure 6:**
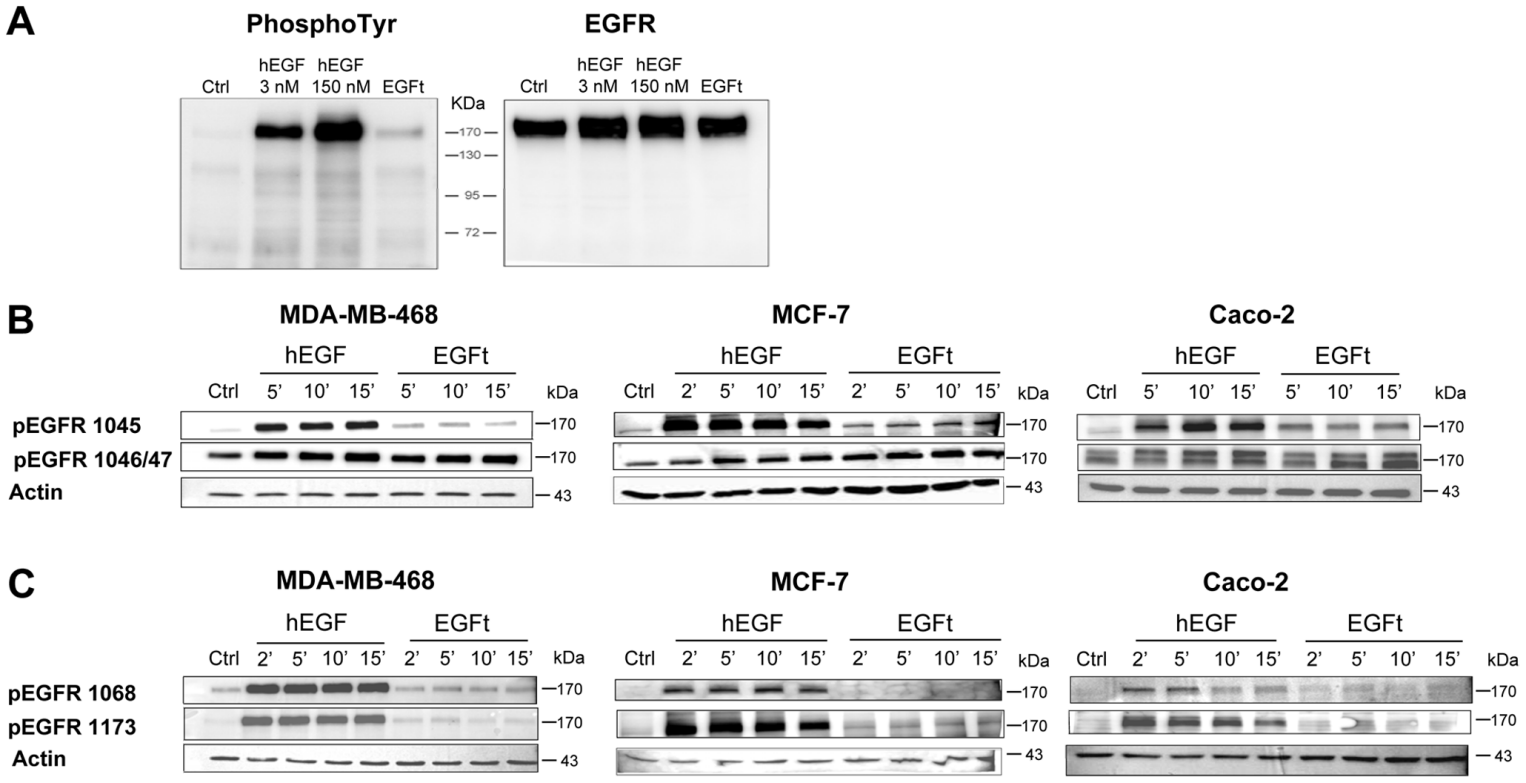
Effect of EGFt on the phosphorylation of EGFR. **A** Analysis of the total phosphorylation of EGFR. MDA-MB-468 cells were treated with 3 nM, 150 nM hEGF or 150 nM EGFt for 10 min. The whole cell lysates were analyzed in parallel by Western blotting with an antibody against total-phosphotyrosine residues (PY20) and with an antibody against EGFR (EGFR 1005). **B** Analysis of the phosphorylation of the C-terminal residues of EGFR involved in the internalization of the receptor. MDA-MB-468, MCF-7 and Caco-2 cells were treated with 150 nM hEGF or 150 nM nM EGFt for the indicated periods of time. The whole cell lysates were analyzed by Western blotting with site-specific antibodies for phospho-EGFR tyrosine 1045 and serines 1046/47. **C** Analysis of the phosphorylation of the C-terminal residues of EGFR involved in the proliferation signaling pathway. MDA-MB-468, MCF-7 and Caco-2 cells were treated with 150 nM hEGF or 150 nM EGFt for the indicated periods of time. The whole cell lysates were analysed by Western blotting with site-specific antibodies for phospho-EGFR tyrosines 1068 and 1173. Untreated cells were used as a negative control (Ctl) and β-actin levels were used as the loading control in Western blotting.

The ability of the recombinant proteins to activate the EGFR was evaluated by analyzing tyrosine residues phosphorylation after hEGF and EGFt cell stimulation. MDA-MB-468 cells were treated with 3 nM, 150 nM hEGF or 150 nM EGFt for 10 min at 37°C. The cell lysates were analyzed in parallel by Western blotting with an antibody against total phosphotyrosines and an antibody against EGFR to identify the bands corresponding to EGFR. Fig. 6A shows that both recombinant proteins were able to activate the receptor. However, the phosphorylation induced by EGFt was markedly lower, even when hEGF concentration was reduced to 3 nM to normalize the binding affinity, indicating an impaired activation of the receptor.

Next, we compared the effect of EGFt and hEGF on the phosphorylation of four EGFR specific residues involved in internalization and proliferation signalling. Fig. 6B shows that specific residues involved in the internalization of the receptor, Y1045 and S1046/47, were highly phosphorylated when MDA-MB-468 cells were stimulated with hEGF. The phosphorylation of Y1046/47 was similar when the cells were stimulated with EGFt, while Y1045 was only slightly activated (Fig. 6B, left panel). Similar results were obtained in MCF-7 and Caco-2 cells (Fig. 6B, central and right panel). On the other hand, while hEGF induced a strong phosphorylation of the tyrosines involved in proliferative signalling pathways, Y1068 and Y1173, EGFt minimally activated them in any assayed cell line (Fig. 6C). In fact, a decrease in a proliferation signal output of EGFR, like MAPK (p-ERK1/2) and PI3K (p-Akt) activation, was observed in EGFt (150 nM nM) compared to hEGF (3 nM or 150 nM) treatment, being these differences more evident in MCF-7 and Caco-2 than in MDA-MB-468 cell lines (Fig. S3). Altogether, the results suggest the induction of EGFR internalization but a less activation of the proliferation signals when treating cells with EGFt.

### Effect of hEGF and EGFt on EGFR internalization

**Figure 7:**
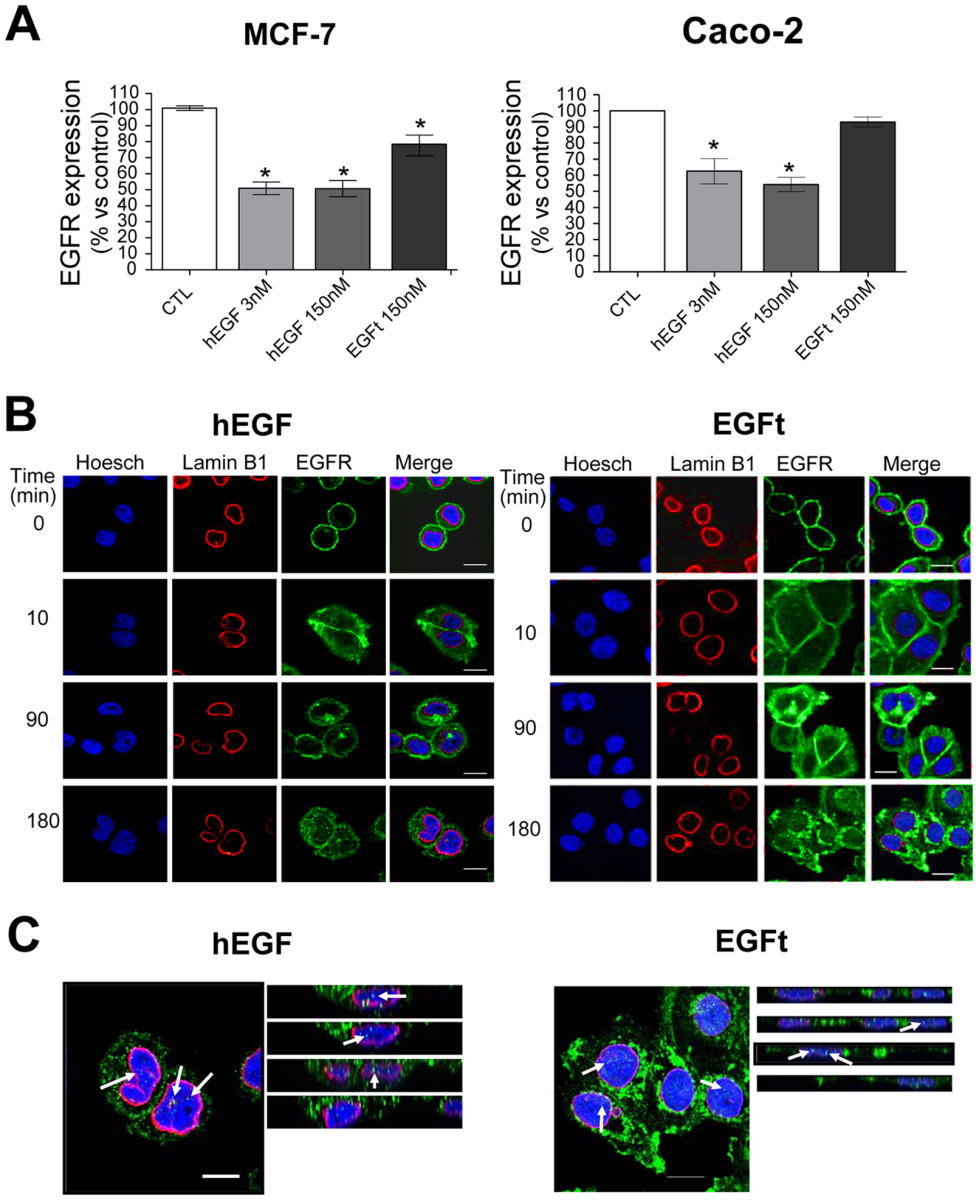
Effect of EGFt on the internalization and localization of EGFR compared to hEGF. A MCF-7 and Caco-2 cells were treated with 3 nM, 150 nM hEGF or 150 nM EGFt for 30 min at 4°C and then incubated at 37°C for 15 min. The detection of EGFR in the cell membrane was determined by performing a cell-ELISA assay with specific antibodies. Each column in the graph represents the relative EGFR expression in the cell membrane versus untreated control cells (CTL) and was the mean ± SEM of three independent experiments (*P<0.05 vs. control cells). B MDA-MB-468 cells were exposed for various times to 150 nM hEGF or 150 nM EGFt and stained for EGFR using FITC anti-EGFR antibody (green). The nucleus and its membrane were stained using Hoescht (blue) and Cy3 anti-lamin B1 antibody (red), respectively. Confocal images were acquired. C The merge images corresponding to 180 min of treatment were magnified and some slices of a merged *xz* reconstruction of the stack (slices at 0.3 microns on z axis) are shown. Arrows indicate green signals within the cell nucleus. Scale bar, 10 μm.

To further explore the ability of EGFt to trigger the internalization of EGFR, we developed a cell-ELISA assay using MCF-7 and Caco-2 cells. In this assay we detected the EGFR that was not internalized and remained on the cell membrane after incubation with either hEGF or EGFt for 15 min at 37°C. Fig. 7A shows the percentage of EGFR on the cell membrane relative to untreated control cells. In MCF-7 cells, EGFR expression on the cell membrane was significantly reduced by 21% by EGFt, while both hEGF treatments induced 50% EGFR internalization. Similar findings were observed in Caco-2 cells regarding to hEGF, which induced 34.5% and 45.6% EGFR internalization at 3 nM and 150 nM, respectively, while the effect of EGFt was more moderate, leading to 6.3% EGFR internalization.

### Effect of hEGF and EGFt on EGFR localization

The cellular localization of EGFR after different times of hEGF or EGFt stimulation was analyzed by immunofluorescent confocal microscopy in MDA-MB-468 cells. Triple labelling in Fig. 7B and 7C shows a similar cellular localization of EGFR in both treatments, hEGF and EGFt respectively. First, in starved condition and before treatments (time 0), EGFR was almost completely located in the plasma membrane. After 10 min with either hEGF or EGFt treatment EGFR were found within the cell near the cell surface. After 90 min, EGFR was observed throughout the whole cytoplasm and plasma membrane in both treatments. Interestingly, after 3 h of treatment a portion of the receptor was observed in several punctuate staining localized in a perinuclear region and some punctuate inside the nucleus.

### Effect of hEGF and EGFt on EGFR degradation

**Figure 8:**
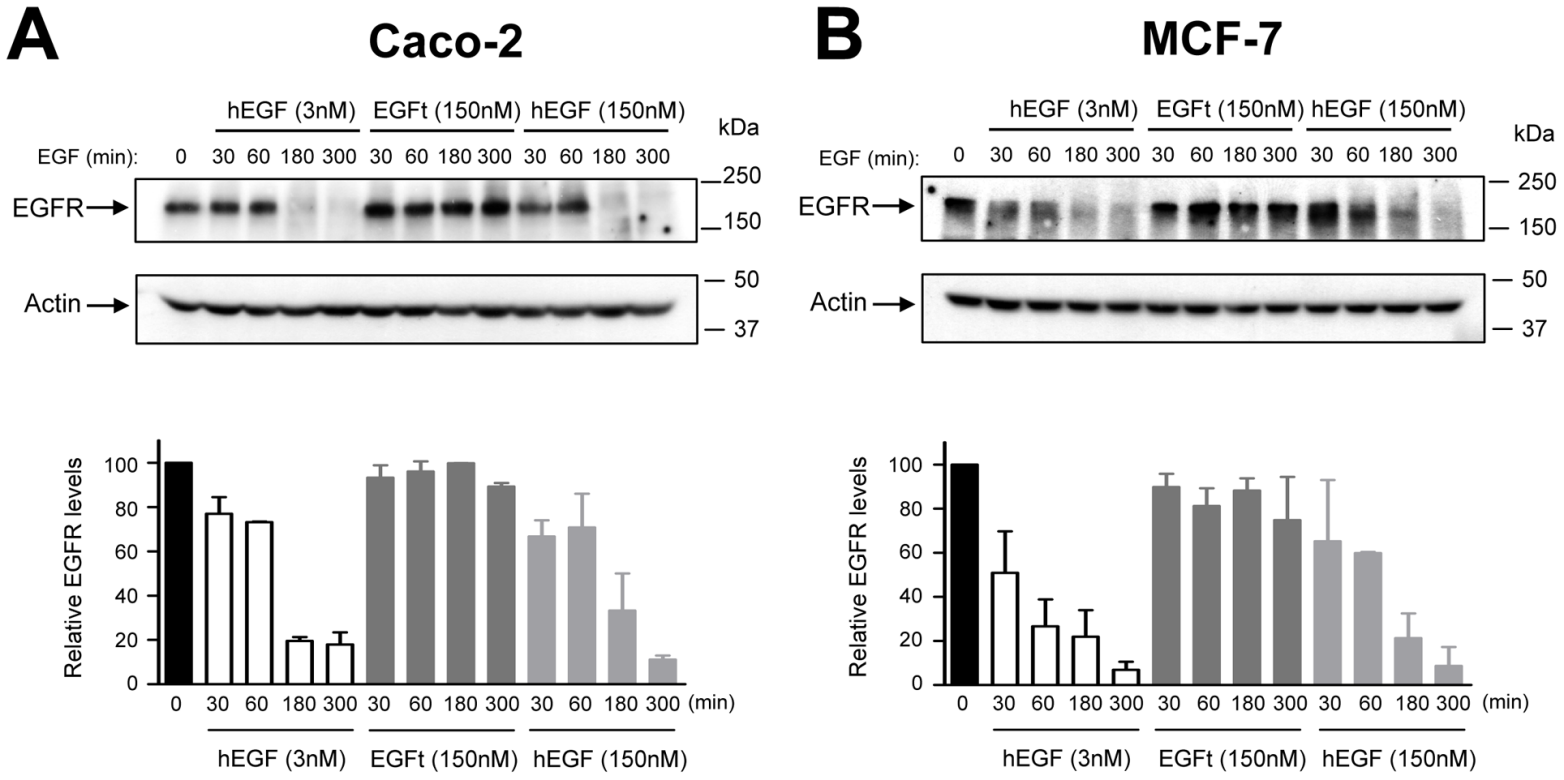
Effect of EGFt treatment on EGFR degradation. A MCF-7 and Caco-2 cycloheximide treated cells were incubated with 3 nM, 150 nM hEGF or 150 nM EGFt at 37°C for different time periods as indicated. Cells were lysed, and the amount of EGFR was determined by Western blotting. Actin levels were used as loading control. B Levels of EGFR were determined by densitometry and normalized versus actin levels. Each column represents the mean of two replicates ± SEM.

The ability of EGFt to trigger the lysosomal degradation of EGFR was also determined. In order to analyze the degradation of the EGFR, MCF-7 and Caco-2 cells were treated with the protein synthesis inhibitor cycloheximide and then the total amount of EGFR was determined by Western blotting after treatment with hEGF or EGFt for 30, 60, 180 and 300 min. As shown in Fig. 8 a great proportion of EGFR was degraded after 180 and 300 min following 3 nM or 150 nM hEGF stimulation. In contrast, close to 100% of the initial receptor level was still intact in all the times analyzed after EGFt stimulation.

### Effect of hEGF and EGFt on cell proliferation

**Figure 9:**
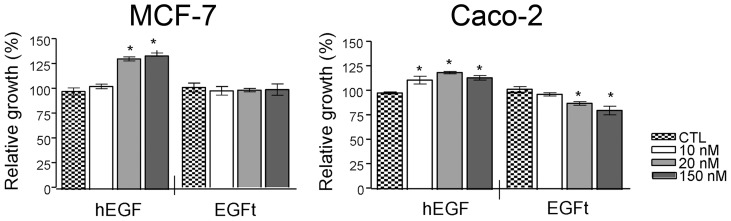
Effect of EGFt on the growth of two human cancer cells. MCF-7 and Caco-2 cells were incubated for 72 h or 96 h h in a culture medium without FBS supplemented with 10, 20 and 150 nM hEGF or EGFt. The cell proliferation was assessed by MTT assays. Each column in the graph represents the relative cell proliferation versus untreated cells (control) and was the mean ± SEM of three independent experiments (*P<0.05 vs. control cells).

We next compared the ability of EGFt and hEGF, to stimulate cell proliferation in two human cancer cell lines, MCF-7 and Caco-2. While hEGF showed a proliferative effect in MCF-7 cells after 3 days of treatment at a concentrations of 20 and 150 nM, EGFt did not significantly modify the proliferation rate at any concentration tested (Fig. 9, left panel). On the other hand, in Caco-2 cells, after 4 days of treatment, hEGF exhibited a moderated but significant proliferative effect at any concentration tested, but EGFt showed a dose-dependent proliferative inhibition (Fig. 9, right panel). These differences between cell lines were probably due to the presence of a potent stimulatory autocrine loop of TGF-α in Caco-2 cells. As Caco-2 cells produce enough TGF-α to sustain their own proliferation, the addition of hEGF did not cause a large increase in proliferation. However, when EGFt was added, it competed with TGF-α for binding EGFR, causing a decrease in cell proliferation.

## Discussion

Signalling through ErbB receptors is of crucial importance in the control of cell proliferation, survival and differentiation. Hence, deregulated signalling by these receptors has been implicated in human malignancies. Recently, EGFR has been found to be a validated target for cancer chemotherapy to treat different tumours. However, the marked differences in the clinical efficiency of different EGFR inhibitors in different carci­nomas, as well as the development of resistance, have limited the efficacy of these drugs [3], [24], [25]. Thus, identifying additional agents that can target EGFR through novel strategies is an important goal in the treatment of cancer.

The main objective of the present work was to produce a peptide with EGFR-blocking activity as anti-tumour agent, based on the PCI, a peptide previously described by our group as an EGFR blocker, but with a lower affinity for EGFR than EGF. In order to design a peptide with higher receptor affinity but preserving the anti-tumour properties of PCI, EGF and PCI structures were superimposed [7], [11], [12]. Interestingly, PCI lacks the C-terminal part of EGF, which is very important for interaction with the receptor. Thus, we hypothesized that the absence of this domain could be responsible for the anti-proliferative activity of PCI. Therefore, we decided to synthesize an EGF analogue without the C-terminal 8 amino acids, preserving the last disulphide bridge (Fig. 1). One of the amino acids removed is Leucine 47, a highly conserved residue, important for EGF binding to the EGFR and its mitogenic activity [26]–[28].

We had previously reported the production of hEGF by refolding it from *E.coli* inclusion bodies [16]. However, the recovery of well folded and bioactive proteins from inclusion bodies involves complicated and costly de-naturation and refolding processes [29]. In the present work we have cloned and expressed both the wild type human EGF and the new truncated EGF peptide (EGFt) using an extracellular expression system that simplifies the purification protocol to only two chromatographic steps and produces the recombinant proteins with their correct folding, a really important feature because the function of this peptide is tightly related to its 3D structure. Furthermore, we have described a novel production methodology by fermentation that increases several times the yield obtained in small batch shake flask production. The enhanced protein production allowed the performance of the *in vitro* assays that addressed the differences between the activation of the EGFR pathway by wild type hEGF and by EGFt in the key EGFR activation steps: receptor binding, dimerization, trans-phosphorylation and internalization.

The affinity of EGFt for EGFR was determined in order to assess if it was increased compared to PCI. As expected, EGFt had a dissociation constant (K_d_) which was lower but in the same order of magnitude as hEGF (nM), while for PCI the effective concentration was one order of magnitude higher (μM) [11], [12]. Thus, the adopted approach significantly increased the affinity of the peptide for the EGFR. Although EGFt showed specific binding and high affinity for EGFR, the affinity of the receptor for EGFt was lower than for the wild type hEGF. This result was also expected because the binding of EGF to its receptor relies mainly on three contact sites (Fig. 1A) and EGFt lacks the Leu47 residue which is of great importance for one of the interactions in site 3 of the EGF-EGFR binding interfaces (Fig. 1B). Binding affinity experiments allowed us to establish the working concentrations for the subsequent experiments.

EGFR dimerization was detected only when cell lysates were activated with hEGF, but not with EGFt. The binding of EGF through the three interaction sites of EGFR shifts the equilibrium between the two distinct EGFR conformations to the untethered one, enabling the dimerization arm in domain II to interact with an identical dimerization arm of another receptor to form the dimer [13], [14]. The differences between dimerization induced by hEGF versus EGFt might be related to the ability to stabilize the untethered configuration. As previously mentioned, EGFt do not interact with all three interaction sites required to promote the dramatic domain rearrangement that will shift the EGFR ectodomain into an untethered configuration in which its dimerization arm is exposed. In agreement, some authors have previously proposed that a ligand that competes effectively with EGF for EGFR binding, but which fails to promote the domain rearrangement, would be a potent antagonist, and also predicted that a monovalent ligand that binds with very high affinity to only domain I or domain III (but not both) would fulfil these criteria [13]. Moreover, recent understanding of the mechanism of action of suramin as a growth factor blocker with anti-cancer activity supports this idea. Suramin binds to EGFR by its C-terminal domain, and the authors suggested that suramin prevents hEGF from interacting with EGFR at site 3 and, as a consequence, EGFR can not undergo the proper conformational change to dimerize and initiate signal transduction [30].

The effects of EGFt in the trans-phosphorylation of EGFR were investigated, assessing the ability of EGFt to initiate downstream signalling pathways. The results indicated that EGFt is able to stimulate EGFR phosphorylation. However, the phosphorylation level induced by EGFt was not as high as hEGF, even at concentrations greater than its K_d_. These results show that even though it is not possible to detect EGFR dimerization, EGFt is still causing EGFR trans-phosphorylation. This indicates that EGFt was likely inducing a small EGFR conformational re-arrangement that could exposes the dimerization arm in part, but probably not sufficient to stabilize the dimer. Consequently, the kinase domain was not fully activated to proper trans-phosphorylate all tyrosine residues.

It has recently been postulated that EGF family members that bind the same receptor are able to stimulate different biological responses both in cell culture and *in vivo*, due to distinctions in the conformation of the ligand-bound receptor and subsequent differences in the sites of receptor tyrosine phosphorylation [31], [32]. Moreover, it has been demonstrated that the structure of the EGFR extracellular domain dimer is distinct when bound to EGF or TGF-α. Thus, ligand-specific differences in the interaction with the receptor may lead to differences in the receptor tyrosine residue availability for the receptor kinase domain [32]. EGFt may induce a distinct EGFR ectodomain conformation than hEGF and, as a consequence, EGFt could be stimulating different downstream responses. Different tyrosine and serine residues were analyzed to address the phosphorylation activation status of two main cellular responses: proliferation and down-regulation of EGFR by internalization of the receptor. Receptor down-regulation is the most prominent regulatory system for EGFR signaling attenuation and involves the internalization and subsequent degradation of the activated receptor in lysosomes. This molecular event mainly involves phosphorylation of Tyr-1045 and Ser-1046/7 of EGFR [33]–[5]. As expected, hEGF strongly induced the phosphorylation of both residues while EGFt did it moderately, indicating that EGFt could be inducing some EGFR internalization thus promoting some EGFR pathway down- regulation. Regarding cell proliferation, signalling by EGFR involves activation of the downstream MAPK/ERK signalling pathway, which is triggered by the phosphorylation of Tyr-1068 and Tyr-1173 [28]. The analysis of the Tyr-1068 and Tyr-1173 phosphorylated residues after activation with either hEGF or EGFt revealed that while hEGF strongly stimulated their phosphorylation, EGFt hardly stimulated them compared to untreated cells. This result indicated that EGFt might not be activating cell growth, a similar result that was observed using PCI [11], [12].

When investigating the effects of EGFt at the cellular level, we found that it was able to induce EGFR internalization, although in a much lesser extent than hEGF, what is in agreement with the reduced phosphorylation of residues involved in down regulation of EGFR. It is known that activated EGFR traffics from the plasma membrane to the cytoplasm through endocytic vesicles. Then, internalized EGFR can be eventually degraded in lysosomes, or recycled to the plasma membrane. Moreover, several reports have identified additional intracellular destinations for the internalized receptor, including the nucleus [36]–[38]. Our confocal microscopy analysis revealed that the localization of the receptor after its activation with either hEGF or EGFt was similar in both cases. Interestingly, after 3 hour of induction, a small amount of EGFR staining was observed into the cell nucleus. Different studies have postulated that intranuclear EGFR may act as a transcriptional co-activator for various oncogenic genes [36], [37]. The study of EGFR degradation revealed that while hEGF stimulated EGFR degradation, EGFt did not. These results suggested that when EGFt stimulated EGFR internalization the receptor was not extensively routed to lysosomes. We hypothesize that these differences in ligand-receptor processing between hEGF and EGFt could be similar to what some authors have observed before with TGF-α. The EGF binding to EGFR is relatively more stable at the pH of endosomes, so upon EGF binding to EGFR it can be transported to lysosomes. In contrast, TGF-α rapidly dissociates from the receptor when exposed to the low pH of endosomes, and the receptor and ligand dissociate and are recycled back to the plasma membrane [39], [40]. If EGFt behaves as TGF-α with respect to internalization, it means that EGFt may be recycled back to the cell surface, where it can exert its blocking activity on the EGFR again. A similar result was observed with PCI [11], [12].

Regarding the effect of EGFt on cell proliferation, we observed that EGFt did not stimulate the proliferation of either tested cell lines. Moreover, a different cell proliferation response was detected between MCF-7 and Caco-2 cell lines treated with EGFt. While in MCF-7 EGFt did not stimulate cell growth at any of the concentrations tested, in Caco-2 cells EGFt inhibited about 20% of cell growth, compared to untreated control cells. Caco-2 cell line has a powerful autocrine loop of TGF-α and its growth depends on it [41]–[43]. These results indicate that EGFt inhibits ligand autocrine mitogenic activity by competing with TGF-α. So that, the data suggest that EGFt is able to inhibit the *in vitro* growth of those cancer cell lines that depend on an autocrine induction of cell proliferation by secretion of EGFR ligands.

In conclusion, we have demonstrated that EGFt binds to EGFR but does not stabilize the dimer formation, leading to an impaired EGFR trans-phosphorylation on tyrosine residues involved in proliferation signalling. Interestingly, EGFt induces the internalization of the receptor and presumably promotes the translocation of the receptor to the cell nucleus. Furthermore, EGFt competes with EGFR native ligands, inhibiting the proliferation of cells with an autocrine growth control via EGFR, acting as an inverse agonist or EGFR blocker. These findings indicate the importance of the hydrophobic interaction of EGF with EGFR on site 3 interface to activate the mitogenic EGFR signalling, and open up the possibility to insert new amino acid changes that could increase EGFt affinity for EGFR and its blocking activity.

Moreover, EGFt could also be used as a targeted toxin delivery agent. In this respect, we are currently developing ^111^In–DTPA–EGFt as a potential Auger electron-emitting radiotherapeutic for EGFR-positive breast cancer. The Auger electrons emitted by ^111^In in the nucleus of EGFR-positive breast cancer cells by ^111^In-DTPA-hEGF were shown to provide potent cytotoxic effects *in vitro* and tumour growth-inhibition *in vivo* [44], [45]. EGFt would have advantages over hEGF for constructing such a radiotherapeutic agent due to its lack of EGFR-mediated growth-stimulatory activity.

## Supporting Information

Figure S1 **Assessment of the optimal cell concentration for cell proliferation assays.** To determine the optimal initial seeding density, different cell concentrations (1000, 2000, 3000, 4000, 5000, 6000, 7000 and 8000 cells per well) were plated into 96-well plates (6 replicates per cell concentration) and incubated as described in the proliferation assay. The cells were allowed to attach and grow for 72 h, starved for 24 h and then treated with the highest concentration of hEGF used in the proliferation assay (150 nM) for 72 h (MCF-7 cells) or 96 h (Caco-2 cells). Finally, the proliferation of the cells was determined by an MTT assay and the main absorvance was represented versus the number of seeded cells. 5000 Caco-2 and 4000 MCF-7 cells/well were selected as optimal initial cell concentrations as they lied within the linear portion of the plot, indicating that cells were still in an exponential growth rate at the end of the experiment.
(TIF)

Figure S2 **Effect of EGFt on the dimerization of EGFR. A** Cell lysates from MDA-MB-468 cells were treated with the indicated concentrations of hEGF, EGFt or a mixture of both for 30 min. Untreated cells were used as control. Then the samples were cross-linked by addition of 40 mM of glutaraldehyde and analyzed by Western blotting using an anti-EGFR antibody. The position of the EGFR monomers and dimers is indicated. **B** To examine the effect of EGFt on EGFR heterodimerization with HER2, MDA-MB-468 cells were treated with 150 nM hEGF, 150 nM EGFt or medium alone as control. After performing the dimerization assay, the samples were analyzed by Western blotting using antibodies against EGFR (left panel) and HER2 (right panel). The position of the EGFR monomers and dimers is indicated.
(TIF)

Figure S3 **Comparative effect between hEGF and EGFt on MAPK and Akt activation in MCF-7, Caco-2 and MDA-MB-468 cells.** Serum starved MCF-7 (A) Caco-2 and MDA-MB-468 (B) cells growth in 6 well plates were stimulated with 3 nM, 150 nM hEGF or 150 nM EGFt as specified for the period of time indicated at 37°C. Lysates with equal amount of protein were electrophoresed and phosphorylated EGFR (p-Tyr), MAPK (p-ERK1/2) and Akt/PKB (p-Thr308) were analyzed by Western blotting. Actin detection was used as a loading control. Figure shows one representative experiment from duplicate samples.
(TIF)
