# Supplementary figures and images for: Development of an Epidermal Growth Factor Derivative with EGFR Blocking Activity
Source: PLoS One. 2013 Jul 30;8(7):e69325. doi: 10.1371/journal.pone.0069325 (PMC3728333; doi:10.1371/journal.pone.0069325)

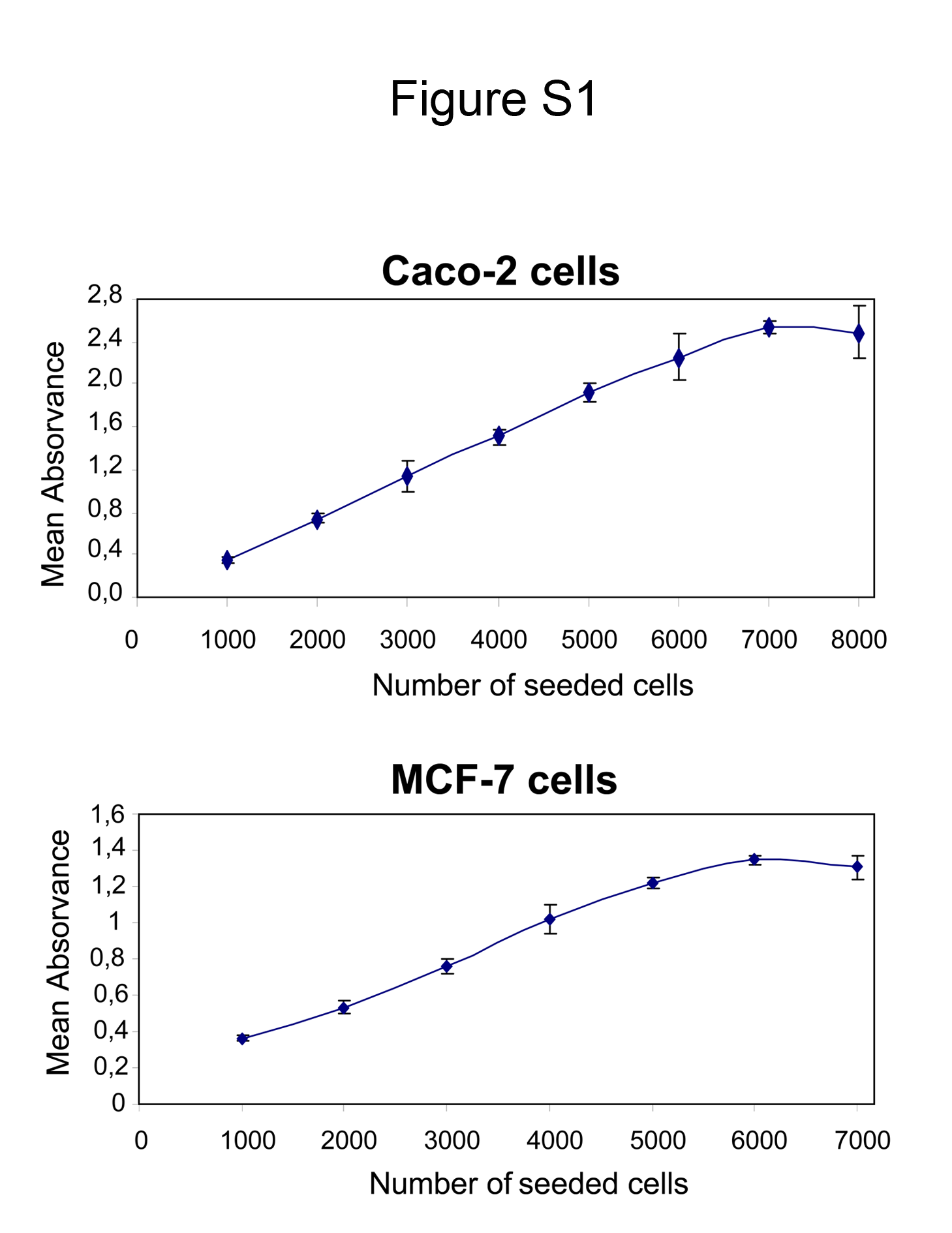

Supplement: Figure S1 — Assessment of the optimal cell concentration for cell proliferation assays. To determine the optimal initial seeding density, different cell concentrations (1000, 2000, 3000, 4000, 5000, 6000, 7000 and 8000 cells per well) were plated into 96-well plates (6 replicates per cell concentration) and incubated as described in the proliferation assay. The cells were allowed to attach and grow for 72 h, starved for 24 h and then treated with the highest concentration of hEGF used in the proliferation assay (150 nM) for 72 h (MCF-7 cells) or 96 h (Caco-2 cells). Finally, the proliferation of the cells was determined by an MTT assay and the main absorvance was represented versus the number of seeded cells. 5000 Caco-2 and 4000 MCF-7 cells/well were selected as optimal initial cell concentrations as they lied within the linear portion of the plot, indicating that cells were still in an exponential growth rate at the end of the experiment. (TIF) [file pone.0069325.s001.tif]

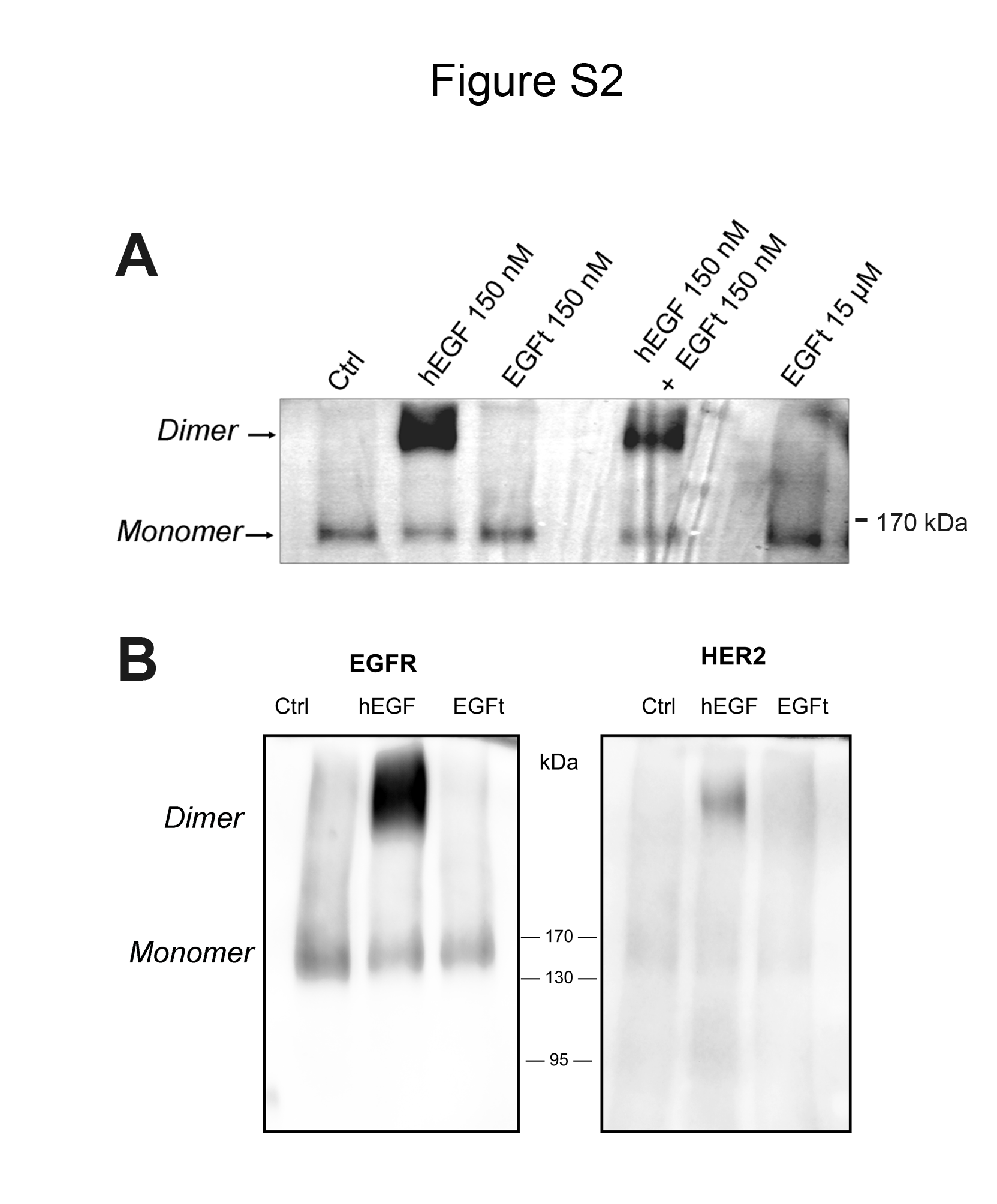

Supplement: Figure S2 — Effect of EGFt on the dimerization of EGFR. A Cell lysates from MDA-MB-468 cells were treated with the indicated concentrations of hEGF, EGFt or a mixture of both for 30 min. Untreated cells were used as control. Then the samples were cross-linked by addition of 40 mM of glutaraldehyde and analyzed by Western blotting using an anti-EGFR antibody. The position of the EGFR monomers and dimers is indicated. B To examine the effect of EGFt on EGFR heterodimerization with HER2, MDA-MB-468 cells were treated with 150 nM hEGF, 150 nM EGFt or medium alone as control. After performing the dimerization assay, the samples were analyzed by Western blotting using antibodies against EGFR (left panel) and HER2 (right panel). The position of the EGFR monomers and dimers is indicated. (TIF) [file pone.0069325.s002.tif]

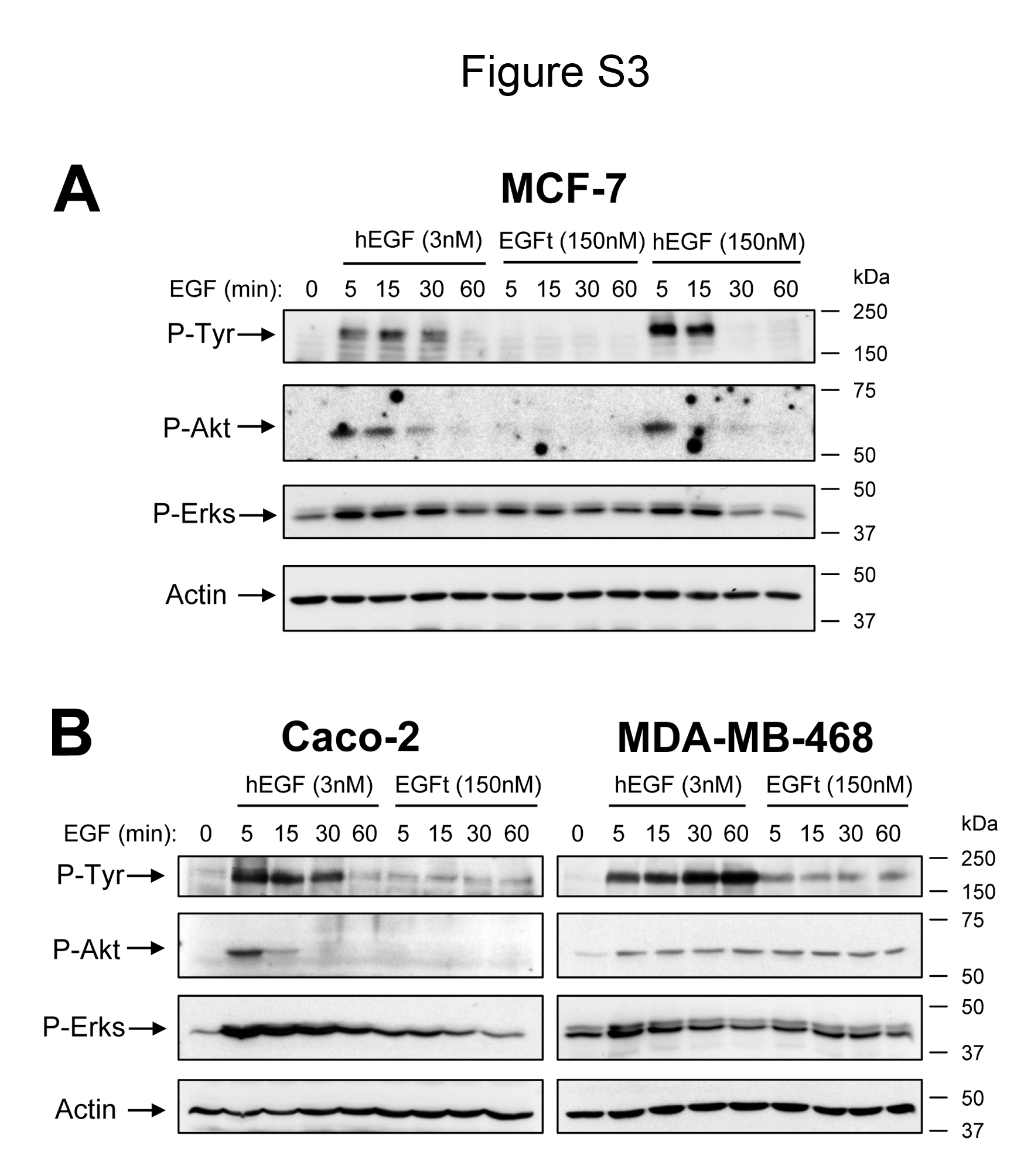

Supplement: Figure S3 — Comparative effect between hEGF and EGFt on MAPK and Akt activation in MCF-7, Caco-2 and MDA-MB-468 cells. Serum starved MCF-7 (A) Caco-2 and MDA-MB-468 (B) cells growth in 6 well plates were stimulated with 3 nM, 150 nM hEGF or 150 nM EGFt as specified for the period of time indicated at 37°C. Lysates with equal amount of protein were electrophoresed and phosphorylated EGFR (p-Tyr), MAPK (p-ERK1/2) and Akt/PKB (p-Thr308) were analyzed by Western blotting. Actin detection was used as a loading control. Figure shows one representative experiment from duplicate samples. (TIF) [file pone.0069325.s003.tif]
